# The implementation of complex infection control bundles to prevent colorectal surgical site infections: a survey of SHEA research network hospitals

**DOI:** 10.1017/ash.2025.183

**Published:** 2025-06-30

**Authors:** Michael Moran, Michele L Zimbric, Michelle Schmitz, Nasia Safdar, Aurora Pop-Vicas

**Affiliations:** 1 Division of Infectious Disease, University of Wisconsin School of Medicine & Public Health, Madison, WI, USA; 2 Department of Medicine, William S. Middleton Memorial Veterans Hospital, Madison, WI, USA; 3 Department of Infection Prevention, UW Health, Madison, WI, USA

## Abstract

**Background:**

Surgical site infections (SSI) result in significant patient morbidity and excess healthcare costs. Colorectal surgeries have the highest SSI risk, as they manipulate the organ with the largest endogenous bioburden. This risk can be mitigated through complex prevention bundles, shown effective at reducing SSI in multiple studies, although little is known about their “real-world” use.

**Methods:**

To obtain further insight into the implementation of SSI prevention bundles consisting of guideline-recommended infection control elements in colorectal surgery, we distributed a multiple-choice survey to the hospitals within the Society for Healthcare Epidemiology of America Research Network from November 2022 to December 2023.

**Results:**

A total of 42 (45%) hospitals completed the survey. The bundle elements most used were intravenous pre-operative antibiotic prophylaxis (88%) and skin prep with an alcohol-chlorhexidine solution (86%). Infection control elements of surgical closure such as glove change and separate instrument tray were reported by 67% and 64%, respectively. Combined oral antibiotics with mechanical bowel prep were reported by 52%. Less than 50% of hospitals reported consistent bundle audit and feedback to frontline surgical staff. The most persistent barriers to implementation were a general culture resistant to change (40%) and clinicians’ lack of compliance with the institutional bundle (38%).

**Conclusions:**

Our study found significant variability in the implementation of bundles consisting of multiple infection control elements to prevent SSI in clinical practice. Further research is needed to determine the strategies most effective in optimizing high-fidelity adoption of complex prevention bundles and to study their effect on SSI in colorectal surgery.

## Introduction

Surgical site infections (SSI) have significant morbidity, healthcare costs greater than $3.3 billion per year in the United States, and prolonged hospitalizations by up to 9.7 days.^1,2^ Colorectal surgeries have the highest incidence of SSI, with rates ranging from 15-30% in some studies.^3–6^ Most hospitals have used the Society for Healthcare Epidemiology of America (SHEA), APIC, IDSA, or CDC clinical guidelines^2,7^ to design multi-element “SSI prevention bundles” to be implemented in the peri-, intra-, and post-operative periods.^8^ However, colorectal SSI persists in the clinical setting. For example, the most recent CDC report on nosocomial infections shows that 11 states still experienced more observed colorectal SSI than predicted (SIR > 1) in 2022, while the national SSI average has not significantly changed compared to prior years.^9^ This lack of progress may be due to “real-world” implementation difficulties for such complex bundles. Therefore, our objectives for this study were to assess the type of bundled infection control elements used for colorectal SSI prevention, and the strategies related to their implementation in the acute care setting.

## Methods

We performed a cross-sectional study using an electronic survey distributed via Qualtrics to hospitals enrolled within the Society for Healthcare Epidemiology Research Network (SRN). The SRN includes a consortium of US and international institutions who participate in collaborative projects related to healthcare epidemiology and antimicrobial stewardship. The SSI survey consisted of 8 multiple choices and/or 5-point Likert scale-type responses assessing annual colorectal surgical volume, infection prevention bundle elements most often used, methods of auditing bundle compliance, perceived importance for each bundle element, and perceptions related to implementation barriers successfully mitigated or still persistent at the time of the survey. The survey was distributed initially on November 20, 2022, to all institutions participating in the SRN at that time, with 2 additional email reminders, each distributed several months apart, until survey closure on December 31, 2023. Institutions that completed < 75% of the survey questions were excluded from the analysis, and duplicate responses were removed. We used STATA SE (StataCorp, College Station, TX) for descriptive and univariate data analysis. Categorical variables were analyzed by the chi-square test, and continuous variables by the Student’s t-test, with a two-sided p value < 0.05 considered statistically significant.

## Results

**Figure 1.**
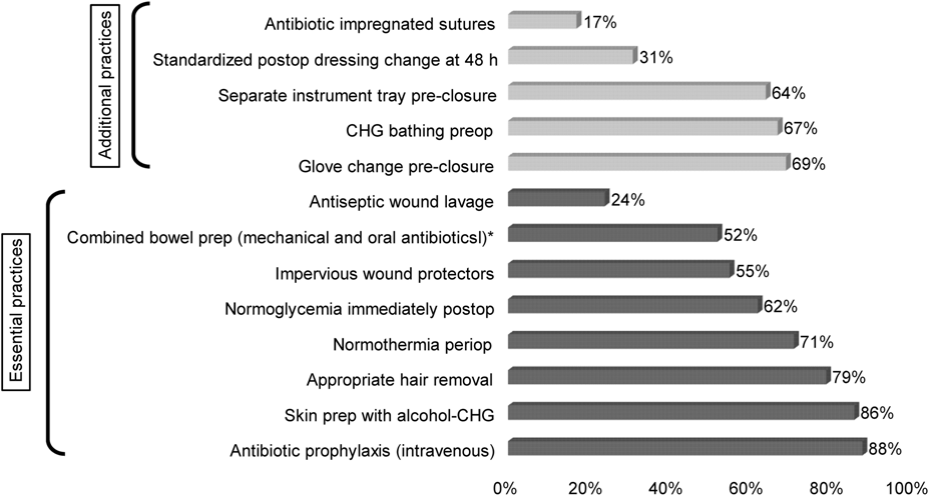
Surgical site infection prevention bundle elements reported as consistently used by participating hospitals. Numbers are reported as a percentage of the total 42 hospitals responding to the survey. Bundle elements deemed “essential practice” by SHEA/IDSA/APIC are shown in dark color; bundle elements additionally recommended in the literature (see references 7, 20) are shown in lighter color. *Updated guidelines recommend combined parenteral and oral antibiotics as essential practice, see discussion for this bundle element.

Of the 93 institutions surveyed, a total of 42 (36 US, 6 international) hospitals provided complete responses and were included in the analysis (45% response rate). Seven participants were excluded from the analysis due to incomplete responses (< 75% of the survey). Baseline characteristics for responding institutions are included in Table 1. Approximately half of the participants (52%) were academic medical centers. Figure 1 shows the SSI prevention bundle elements reported as consistently used by the hospital survey participants. The most frequently used elements were intraoperative antibiotic prophylaxis and intraoperative skin preparation using alcohol-based chlorhexidine, used by 88% and 86% of hospitals, respectively. There were no statistically significant differences in the use of specific bundle elements among hospitals with high versus medium or low surgical volume.

**Table 1.** Baseline characteristics of the 42 hospitals who completed the survey

Institution type	N (%)
Academic medical center	22 (52)
Community teaching hospital	13 (31)
Community non-teaching hospital	3 (7)
Other (VA, Public, outpatient surgical center)	4 (10)
**Annual colorectal surgical volume**	
Low: < 250	11 (26)
Medium: 250 - 500	21 (50)
High: > 500	10 (24)

Table 2 shows the proportion of hospitals that performed audits on at least a yearly basis as an effort to enhance compliance with SSI bundle elements. Intraoperative antibiotic prophylaxis and skin prep with an alcohol-chlorhexidine-containing solution were audited most frequently (71% and 52% of the hospitals respectively), while all other bundle elements were consistently audited in less than 50% of the hospitals. Feedback to frontline clinicians on bundle adherence was performed most frequently for intraoperative antibiotic prophylaxis (57% of the hospitals), while consistent feedback for all other bundle elements was reported by less than 40% of the hospitals. Most hospitals (71%) used the electronic health record to audit compliance, while 33% of the hospitals also reported direct observations as a method for auditing compliance. Hospitals with higher surgical volume were more likely to audit compliance with correct antibiotic prophylaxis (*P* = 0.04) and use of combined mechanical bowel prep with oral antibiotics (*P* = 0.02).

**Table 2.** Percent of hospitals who reported conducting audit and feedback for adherence to certain surgical site infection prevention bundle element on at least a yearly basis. Numbers are reported as a percentage of a total of 42 hospitals responding to the survey

SSI Prevention Bundle Element	Audited	Feedback
Intraoperative antibiotic prophylaxis	71%	57%
Skin prep with chlorhexidine-alcohol-containing solution	52%	33%
Maintaining intraoperative normothermia	48%	33%
Maintaining intraoperative normoglycemia	43%	31%
Appropriate hair removal	36%	21%
Glove change prior to surgical closure	31%	19%
Separate instrument tray for surgical closure	29%	19%
Combined mechanical bowel prep with oral antibiotics	21%	19%
Standardized wound dressing change at 48 h postop	12%	7%

Table 3 shows perceived barriers to bundle implementation—successfully mitigated or still persistent—at the time of the survey. An institutional culture resistant to change and the absence of consistent auditing and feedback were perceived as the most common persistent barriers by 40% and 33% of hospitals, respectively.

**Table 3.** Perceived barriers to surgical site infection (SSI) prevention bundle implementation. Numbers are reported as a percentage of a total of 42 hospitals responding to the survey

Barriers to bundle implementation	Successfully mitigated	Persistent
Lack of clinicians’ compliance with bundle	49%	38%
Lack of clinicians’ knowledge regarding bundle	38%	24%
Inadequate audit and feedback for bundle compliance	38%	33%
Skepticism about bundle effectiveness	29%	36%
Supplies and equipment not easily available	26%	7%
Patient difficulty with bundle compliance at home	26%	29%
Inadequate institutional leadership support for SSI prevention	26%	19%
Not enough automatic reminders or computerized orders	19%	21%
General culture resistant to change	24%	40%
Insufficient patient education with bundle	21%	14%
Inadequate supervision to ensure compliance	12%	19%
Not enough visual aids or reminders	10%	12%

Institutions were asked to rank the bundle elements felt to be most important for SSI prevention in colorectal surgery on a 5-point Likert scale. Intraoperative antibiotic prophylaxis was ranked as either “most important” or “very important” by 76% of participants; intraoperative skin prep with alcohol-CHG containing solution by 52%; combined mechanical bowel prep with oral antibiotics by 33%; and patient preoperative bathing with chlorhexidine by 26% of hospitals participating in the survey.

## Discussion

The surgical infection control literature shows colorectal bundles to be effective in SSI prevention. A 2020 meta-analysis found an overall SSI reduction of 44%, with complex, ≥ 11 element-bundles consisting of both traditional and newer interventions demonstrating the greatest SSI reduction.^10^ Our survey suggests variable success in the implementation of guideline-recommended SSI prevention bundle elements in acute care hospitals. Intra-operative antibiotic prophylaxis and skin prep with alcohol-chlorhexidine solutions, recognized as very important elements of SSI prevention, were reported as consistently used by most participating hospitals. On the other hand, mechanical bowel prep combined with oral antibiotics was reported to be consistently used by only half of the participating hospitals and was considered important by less than one-third of participants. Perhaps this finding reflects reticence in fully adopting an intervention that has been a debated topic in the surgical literature. The controversy has primarily centered around the use of mechanical bowel prep, viewed by some experts as minimally effective,^11^ while associated with potential abdominal discomfort and dehydration for some patients. However, in combination with oral antibiotics, mechanical bowel prep has been shown to reduce SSI, postoperative ileus, anastomotic leak, and 30-day mortality in colorectal surgery by multiple studies.^12–14^ The recent MOBILE2 randomized clinical trial, conducted to help clarify this dispute, reinforced the effectiveness of mechanical bowel prep combined with oral antibiotics in preventing SSI and anastomotic dehiscence in surgeries involving rectal resection.^15^ There is a growing consensus, emphasized in the most recent updated guidelines^7^ that the essential practice for prevention is the combination of oral and intravenous antibiotics rather than the mechanical bowel prep component,^16,17^ which by itself has not been shown effective in randomized trials.^18,19^ Further research is needed to elucidate the form of bowel prep that is most effective in preventing SSI in colorectal surgery.

Expert consensus in colorectal surgery also supports the use of a mini bundle dedicated to incision closure (sterile tray, pre-closure glove change, antimicrobial sutures), besides impervious wound protectors/retractors and antiseptic wound lavage.^20^ Our survey indicates that many hospitals have implemented some of these elements (pre-closure glove change 69%, separate instrument tray 63%, wound edge protectors or retractors 55%), although there is room for improvement. The use of antimicrobial-impregnated sutures was low, which may reflect cost constraints in some settings.

Our study found that few hospitals monitored bundle adherence through routine audit and feedback, which is recommended as an essential practice in SSI prevention.^7^ Audit and feedback has long been recognized as an effective strategy in changing professional practice, especially when delivered frequently in settings with low adherence,^21^ and has been associated with significant reduction in SSI rates.^22^ In colorectal surgery, audit and feedback are generally well regarded, being perceived as important in increasing individual awareness and accountability and helpful in adopting recommended SSI prevention interventions.^23^

Of course, successfully implementing complex SSI prevention bundles in clinical practice requires a multifaceted, multidisciplinary approach,^24,25^ preferably under the leadership of a surgeon-champion.^26,27^ While audit and feedback have been cited as the most frequently used strategy, organizational culture, monitoring the performance of healthcare delivery, using reminders, and increasing education have also been recognized among the “top five” implementation strategies to prevent SSI.^28^ Employed together in a multi-modal implementation approach, these strategies were associated with a 52% risk reduction in SSI, as reported in the systematic review by Tomsic *et al*.^28^ In our study, respondents identified lack of audit and feedback, need to change organizational culture, and need for more educational support as persistent implementation barriers in 25%–40% of the participating hospitals, underscoring the need for further work in improving these aspects of SSI prevention bundle implementation.

Our study has several limitations. Since the survey was anonymous, we do not have data on the colon SSI rates for each participating hospital and cannot analyze how any of the bundle elements and implementation strategies studied are correlated with SSI prevention. The lower survey response rate and the survey distribution to only hospitals participating in the SRN are shortcomings, as the findings may not be generalizable to all acute care settings. In addition, the limitations inherent to survey studies, such as potential for response bias, inability to verify the accuracy of self-reported data, and difficulty capturing the complexity of the implementation of SSI prevention through discrete survey questions apply here as well.

In conclusion, our study highlights variability in the implementation of complex SSI prevention bundles in acute care hospitals. Further research is essential to refine the strategies needed to optimize the effectiveness of these bundles by increasing their adoption in the clinical setting.

## References

[1] Zimlichman E , et al. Health care-associated infections: a meta-analysis of costs and financial impact on the US health care system. JAMA Intern Med 2013;173:2039–2046. 23999949 10.1001/jamainternmed.2013.9763

[2] Berrios-Torres SI , et al. Centers for disease control and prevention guideline for the prevention of surgical site infection, 2017. JAMA Surg 2017;152:784–791. 28467526 10.1001/jamasurg.2017.0904

[3] Dixon LK , et al. Surgical site infection prevention bundle in elective colorectal surgery. J Hosp Infect 2022;122:162–167. 35151765 10.1016/j.jhin.2022.01.023

[4] Hubner M , et al., Surgical site infections in colon surgery: the patient, the procedure, the hospital, and the surgeo*n* . Arch Surg 2011;146:1240–1245. 21768407 10.1001/archsurg.2011.176

[5] Tanner J , et al. Post-discharge surveillance to identify colorectal surgical site infection rates and related costs. J Hosp Infect 2009;72:243–250. 19446918 10.1016/j.jhin.2009.03.021

[6] Smith RL , et al. Wound infection after elective colorectal resection. Ann Surg 2004;239:59–605; discussion 605-7. 10.1097/01.sla.0000124292.21605.99 PMC1356267 15082963

[7] Calderwood MS , et al. Strategies to prevent surgical site infections in acute-care hospitals: 2022 Update. Infect Control Hosp Epidemiol 2023;44:695–720. 37137483 10.1017/ice.2023.67 PMC10867741

[8] Anderson DJ , et al., Strategies to prevent surgical site infections in acute care hospitals: 2014 update. Infect Control Hosp Epidemiol 2014;35:S66–88. 25376070 10.1017/s0899823x00193869

[9] *Centers for Disease Control and Prevention* . *Current HAI Progress Report*. Available at: https://www.cdc.gov/healthcare-associated-infections/php/data/progress-report.html#cdc_report_pub_study_section_3-data-tables; last accessed December 24, 2024.

[10] Pop-Vicas AE , et al. Colorectal bundles for surgical site infection prevention: A systematic review and meta-analysis. Infect Control Hosp Epidemiol 2020;41:805–812. 32389140 10.1017/ice.2020.112

[11] Liu, S. , et al., Is mechanical bowel preparation mandatory for elective colon surgery? A systematic review and meta-analysis. Langenbecks Arch Surg 2024;409:99. 38504007 10.1007/s00423-024-03286-z

[12] Toh, J.W.T. , et al. Association of mechanical bowel preparation and oral antibiotics before elective colorectal surgery with surgical site infection: a network meta-analysis. JAMA Netw Open 2018;1:e183226. 30646234 10.1001/jamanetworkopen.2018.3226 PMC6324461

[13] Rollins KE , et al. The role of oral antibiotic preparation in elective colorectal surgery: a meta-analysis. Ann Surg 2019;270:43–58. 30570543 10.1097/SLA.0000000000003145 PMC6570620

[14] Scarborough JE , et al. Combined mechanical and oral antibiotic bowel preparation reduces incisional surgical site infection and anastomotic leak rates after elective colorectal resection: an analysis of colectomy-targeted ACS NSQIP. Ann Surg 2015;262:331–337. 26083870 10.1097/SLA.0000000000001041

[15] Koskenvuo L , et al. Morbidity after mechanical bowel preparation and oral antibiotics prior to rectal resection: the MOBILE2 randomized clinical trial. JAMA Surg 2024;159: 606–614. 38506889 10.1001/jamasurg.2024.0184 PMC10955353

[16] Rybakov E , et al. Impact of oral antibiotic prophylaxis on surgical site infection after rectal surgery: results of randomized trial. Int J Colorectal Dis 2021;36:323–330. 32984909 10.1007/s00384-020-03746-0

[17] Lee JH , et al. Mechanical bowel preparation combined with oral antibiotics in colorectal cancer surgery: a nationwide population-based study. Int J Colorectal Dis 2021;36:1929–1935. 34089359 10.1007/s00384-021-03967-x

[18] Nelson RL , Hassan M. , and Grant MD , Antibiotic prophylaxis in colorectal surgery: are oral, intravenous or both best and is mechanical bowel preparation necessary? Tech Coloproctol 2020;24:1233–1246. 32734477 10.1007/s10151-020-02301-x

[19] Rollins KE , Javanmard-Emamghissi H , and Lobo DN , Impact of mechanical bowel preparation in elective colorectal surgery: a meta-analysis. World J Gastroenterol 2018;24: 519–536. 29398873 10.3748/wjg.v24.i4.519 PMC5787787

[20] Ruiz-Tovar J , et al. Delphi consensus on intraoperative technical/surgical aspects to prevent surgical site infection after colorectal surgery. J Am Coll Surg 2022;234:1–11. 35213454 10.1097/XCS.0000000000000022 PMC8719508

[21] Jamtvedt G , et al. Audit and feedback: effects on professional practice and health care outcomes. Cochrane Database Syst Rev 2006;CD000259. 16625533 10.1002/14651858.CD000259.pub2

[22] Manivannan B , et al. Surveillance, auditing, and feedback can reduce surgical site infection dramatically: toward zero surgical site infection. Surg Infect (Larchmt) 2018;19: 313–320. 29480742 10.1089/sur.2017.272

[23] Nessim C , et al. Surgical site infection prevention: a qualitative analysis of an individualized audit and feedback model. J Am Coll Surg 2012;215:850–857. 23164141 10.1016/j.jamcollsurg.2012.08.007

[24] Hatharaliyadda B , et al. Surgical site infection prevention using “strike teams”: the experience of an academic colorectal surgical department. J Healthc Qual 2024;46:22–30. 38166163 10.1097/JHQ.0000000000000412

[25] Lin, F. , et al. Evaluating the implementation of a multi-component intervention to prevent surgical site infection and promote evidence-based practice. Worldviews Evid Based Nurs 2020;17:193–201. 32282120 10.1111/wvn.12436

[26] Pop-Vicas AE , et al. Surgeons’ mental models of surgical site infection: Insights into adherence with complex prevention bundles. Infect Control Hosp Epidemiol 2022;43: 1249–1255. 33985608 10.1017/ice.2021.161

[27] Massimo Sartelli FC , Abu-Zidan FM , et al. Hey surgeons! It is time to lead and be a champion in preventing and managing surgical site infections! World J Emerg Surg 2020;15. 10.1186/s13017-020-00308-1 PMC7168830 32306979

[28] Tomsic I , et al. Implementation interventions in preventing surgical site infections in abdominal surgery: a systematic review. BMC Health Serv Res 2020;20:236. 32192505 10.1186/s12913-020-4995-z PMC7083020

